# Supplementary material for: Smartphone-operated affordable PCR thermal cycler for the detection of antimicrobial resistant bacterial genes
Source: PLOS Glob Public Health. 2023 Feb 27;3(2):e0001120. doi: 10.1371/journal.pgph.0001120 (PMC10021165; doi:10.1371/journal.pgph.0001120)
Supplement: S1 Text — Table A: Antibiotic susceptibility test for E. coli isolates. Table B: Prevalence of blaCTX-M and blaTEM genes. (DOCX) [file pgph.0001120.s001.docx]

**Smartphone-controlled low-cost thermocycler for the detection of extended spectrum beta-lactamases producing bacterial genes**

**Supplementary information 1**

Table A: Antibiotic susceptibility test for *E. coli* isolates

| Antibiotic Classes | Antibiotics | Antibiotic Concentration  (µg) | Sensitive  % | Intermediate  % | Resistant  % |
| --- | --- | --- | --- | --- | --- |
| β-Lactam/  β-Lactamase inhibitors | Amoxicillin  (AMX) | 10 | 12.9 | - | 87.1 |
|  | Amoxycillin/ Clavulanic acid  (AMC) | 20/10 | 14.8 | - | 85.2 |
|  | Piperacillin/ Tazobactam  (PIT) | 100/10 | 87.1 | - | 12.9 |
| Third generation cephalosporins | Ceftazidime  (CAZ) | 30 | 55.6 | 3.7 | 40.7 |
|  | Ceftriaxone  (CTR) | 30 | 50.1 | 3.6 | 46.3 |
|  | Cefotaxime  (CTX) | 30 | 31.5 | 7.4 | 61.1 |
| Monobactams | Aztreonam  (AT) | 30 | 61.1 | 3.7 | 35.2 |
| Aminoglycosides | Gentamicin  (GEN) | 10 | 83.3 | - | 16.7 |
|  | Amikacin  (AK) | 30 | 90.7 | - | 9.3 |
| Folate inhibitor | Cotrimoxazole  (COT) | 25 | 50.1 | 1.8 | 48.1 |
| Fluoroquinolone | Ofloxacin  (OF) | 5 | 61.1 | 1.8 | 37.1 |
| Quinolone | Nitrofurantoin  (NIT) | 300 | 74.1 | 3.7 | 22.2 |

Table B: Prevalence of *bla*_CTX-M_ and *bla*_TEM_ genes.

| S. N | Sample code | *bla*_CTX-M_ | | *bla*_TEM_ | |  |
| --- | --- | --- | --- | --- | --- | --- |
| 1. | 159388 | - | | - | |  |
| 2. | 155602 | + | | + | |  |
| 3. | 4991 | + | | - | |  |
| 4. | 1555 | + | | + | |  |
| 5. | 158944 | + | | + | |  |
| 6. | 156550 | - | | - | |  |
| 7. | 15546 | + | | - | |  |
| 8. | 158871 | + | | + | |  |
| 9. | N100 | + | | - | |  |
| 10. | 158564 | + | | - | |  |
| 11. | 1512123 | - | | - | |  |
| 12. | 2184112 | + | | + | |  |
| 13. | 693/25 | + | | + | |  |
| 14. | N107 | + | | + | |  |
| 15. | 159194 | + | | - | |  |
| 16. | 354197 | + | | - | |  |
| 17. | 154106 | + | | + | |  |
| 18. | 349756 | - | | + | |  |
| 19. | 155624 | + | | - | |  |
| 20. | 161572 | - | | + | |  |
| 21. | N109 | - | | + | |  |
| 22. | 1406/LF | + | | + | |  |
| 23. | 160938 | - | | - | |  |
| 24. | N110 | + | | + | |  |
| 25. | 158853 | + | | + | |  |
| Total | | | 18 | | 14 | |
